# Unlocking hidden potential: advancements, approaches, and obstacles in repurposing drugs for cancer therapy

**DOI:** 10.1038/s41416-023-02502-9

**Published:** 2023-11-27

**Authors:** Freya R. Weth, Georgia B. Hoggarth, Anya F. Weth, Erin Paterson, Madeleine P. J. White, Swee T. Tan, Lifeng Peng, Clint Gray

**Affiliations:** 1 Gillies McIndoe Research Institute, Newtown, Wellington 6021 New Zealand; 2 Centre for Biodiscovery and School of Biological Sciences, Victoria University of Wellington, Kelburn, Wellington 6021 New Zealand; 3 Wellington Regional Plastic, Maxillofacial & Burns Unit, Hutt Hospital, Lower Hutt, 5040 New Zealand; 4 Department of Surgery, The Royal Melbourne Hospital, The University of Melbourne, Melbourne, VIC 3010 Australia

**Keywords:** Targeted therapies, Industry, Drug regulation, Clinical trials, Health care economics

## Abstract

High rates of failure, exorbitant costs, and the sluggish pace of new drug discovery and development have led to a growing interest in repurposing “old” drugs to treat both common and rare diseases, particularly cancer. Cancer, a complex and heterogeneous disease, often necessitates a combination of different treatment modalities to achieve optimal outcomes. The intrinsic polygenicity of cancer, intricate biological signalling networks, and feedback loops make the inhibition of a single target frequently insufficient for achieving the desired therapeutic impact. As a result, addressing these complex or “smart” malignancies demands equally sophisticated treatment strategies. Combinatory treatments that target the multifaceted oncogenic signalling network hold immense promise. Repurposed drugs offer a potential solution to this challenge, harnessing known compounds for new indications. By avoiding the prohibitive costs and long development timelines associated with novel cancer drugs, this approach holds the potential to usher in more effective, efficient, and cost-effective cancer treatments. The pursuit of combinatory therapies through drug repurposing may hold the key to achieving superior outcomes for cancer patients. However, drug repurposing faces significant commercial, technological and regulatory challenges that need to be addressed. This review explores the diverse approaches employed in drug repurposing, delves into the challenges faced by the drug repurposing community, and presents innovative solutions to overcome these obstacles. By emphasising the significance of combinatory treatments within the context of drug repurposing, we aim to unlock the full potential of this approach for enhancing cancer therapy.

**Figure:**
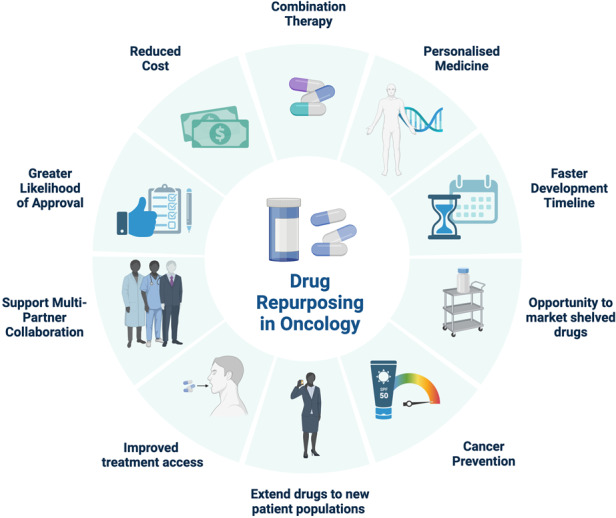
The positive aspects of drug repurposing in oncology are underscored here; encompassing personalized treatment, accelerated development, market opportunities for shelved drugs, cancer prevention, expanded patient reach, improved patient access, multi-partner collaborations, increased likelihood of approval, reduced costs, and enhanced combination therapy.

The positive aspects of drug repurposing in oncology are underscored here; encompassing personalized treatment, accelerated development, market opportunities for shelved drugs, cancer prevention, expanded patient reach, improved patient access, multi-partner collaborations, increased likelihood of approval, reduced costs, and enhanced combination therapy.

## Introduction

Drug repurposing, also known as drug repositioning, reprofiling, reusing, and rediscovery, is the process by which a known drug or compound is used for a new indication [1]. Its use in cancer therapy dates back to the very first chemotherapeutics, which arose from research on the observed anti-tumour potential of mustard gas on skin cancers [2–4]. More recently, interest in repurposed drugs has increased as a potential counter to the ever-increasing cost, low approval rates and prolonged time to market of novel drugs. The increasing opportunity for collaboration, as well as opportunities for funding, make drug repurposing more viable in this climate. There is currently an important role played by philanthropy, governments/states and not-for-profit organisations in early phase drug repurposing. A few current institutions, initiatives and funds with goals of furthering the development of repurposed drugs are; The National Center for Advancing Translational Sciences, The Wellcome Trust Health Innovation Challenge Fund, European Infrastructure for Translational Medicine, The Broad Institutes Drug Repurposing Hub, Cures Within Reach, Repurposing Drugs in Oncology (reDO) Project, and The Structural Genomics Consortium [5]. Multi-partner collaborations such as these, and those between academia and industry, have the opportunity to make drug repurposing more feasible.

The incidence of cancer is increasing, as is the resultant economic burden of cancer treatment on countries’ health systems. Between 1990 and 2013, there was a 75.6% increase in the number of cases of cancer globally, with 35%, 35.6% and 5% of this change attributable to population growth, the aging population, and a change in incidence rates [6]. Alongside the increase in demand for cancer treatment, there has also been a steady increase in the cost of treating each patient, such that health spending on cancer care has outpaced the incidence of cancer [7]. The median cost of cancer treatment at the time of the United States of America (USA) Food and Drug Administration (FDA) or the European Medicines Agency approval has increased from less than $100 per month in the 1990s to approximately $10,000 per month in 2011. Once adjusted for inflation and health benefits, the average price of anti-cancer drugs at the time of launch has increased by 10% annually between 1995 and 2013—an average increase of $8500 per year [8, 9]. A corresponding increase in the public health systems’ budgets spent on cancer treatment has been observed in Europe (EU-27, plus Iceland, Norway, Switzerland, and the United Kingdom), the total health expenditure on cancer care increased by 98%, from €52 billion in 1995 to €103 billion in 2018. The amount spent specifically on cancer drugs more than tripled from €10 billion in 2005 to €32 billion in 2018. Cancer drug treatment costs were found to be even higher in the USA, with a median cost 2.31 times higher than Europe, and the financial burden is more often borne by individuals and private insurance companies than the government [7, 10, 11]. In a New Zealand (NZ) context, despite a remarkable track record for restraining public pharmaceutical expenditure, the drug purchase agency PHARMAC’s funding has increased by 25% over the four years to March 2022, in a large part, to fund more cancer drugs publicly [12, 13]. Global cancer drug sales are projected to increase even further in the next few years, from $193 billion USD in 2022 to $377 billion USD by 2027, with the drivers including earlier cancer diagnoses and decreased mortality resulting in longer treatment periods, as well as increased access to novel cancer drugs in more parts of the world, and the continued influx of new drugs to the market [14].

The number of novel FDA-approved drugs per billion US dollars invested in research and development (R&D) has halved every nine years since 1950, and the likelihood of approval (LOA) for cancer drugs in phase I clinical trials is only 6.7%, the lowest LOA of any drug type, and about half the LOA of non-oncology drugs [15, 16]. This exponential decrease, termed the ‘Eroom’s Law’, has occurred despite major advancements in the technology and scientific knowledge used in modern drug discovery [15]. Scannell et al. propose four main causes for the Eroom’s Law: (1) any new drug invented now must offer significantly improved health outcomes compared to the cheaper drugs already available, in order to gain a foothold in the market; (2) there are more regulatory hurdles in today’s drug development which require more resources to gain approval; (3) there is a tendency to invest in R&D, to be the first in the more lucrative position of launching a new type of drug; and (4) the implication that, in order for the Eroom’s law to have become a problem, the industry must not have adopted R&D methods as advanced as commonly assumed, i.e., we have, to some extent, industrialised the wrong set of R&D activities [15].

An often overlooked and underappreciated solution to many of these increasingly pressing issues is the repurposing of drugs for a new indication in cancer treatment. Drugs which can be repurposed include; generic (off-patent) drugs already available on the market; on-patent drugs, including those still undergoing clinical trials; and failed drugs for the original indication. Drugs which have the potential to be repurposed for cancer treatment may have originally been cancer drugs used for a different cancer, or non-oncology drugs used for a different indication altogether.

**Fig. 1:**
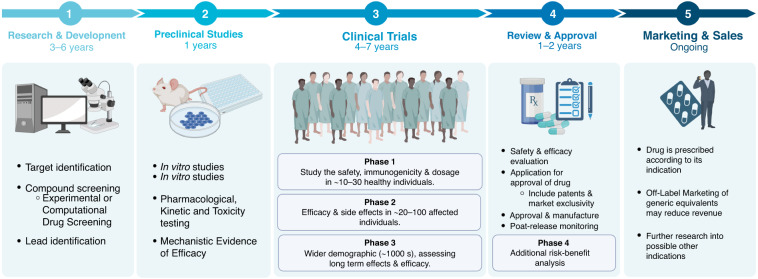
A common drug development timeline [161]. Repurposed drugs can often join this timeline in the third stage (clinical trials) skipping up to 7 years of study that a new drug would require. Created using Biorender, May 2023.

Repurposing a drug for a new indication is generally cheaper and faster than developing an entirely new drug. The preclinical and phase I clinical trials, which assesses a drug’s safety and tolerance, are already complete in the initial development process, so while it takes approximately 13 to 15 years and costs around US$2–3 billion to bring a novel drug to the market, repurposing a drug is estimated to take only 6.5 years and cost an average of $300 million [17, 18] (Fig. 1). Later-phase clinical trials for the repurposed drug for its new indication, the regulatory approval process and any reformulation required, will cost much the same as for a novel drug. However, repurposed drugs are less likely to fail overall in comparison to novel drugs development [19]. Another benefit of having previously undergone the early phase clinical trials is that safety, dosing, and pharmacokinetic data is already available for repurposed drugs [20].

Cancer, with its inherent heterogeneity, rapid development of treatment resistance, and the intricate web of cellular pathways that contribute to its malignancy, presents a challenge for single-agent therapies. It is increasingly evident that the future of effective cancer treatment lies in the realm of combinatory therapies. This article is dedicated to exploring the promise of combinatory approaches, recognising their potential to tackle the multifaceted nature of cancer and improve treatment outcomes. In this review, we aim to shed light on the potential of drug repurposing, with a particular emphasis on promising drugs identified in vitro. It is important to note that while these drugs exhibit significant potential in pre-clinical studies, their performance in clinical settings may vary. We acknowledge that not all drugs discussed here may ultimately prove effective in combinatory cancer therapies, and there are indeed numerous instances of unsuccessful trials, especially of single-agent drugs in this field.

## Repurposed drugs

### Repurposed drug classification

In this review, we adopt a comprehensive view of drug repurposing. It encompasses not only dramatic changes in drug applications but also more subtle adaptations that reveal previously unnoticed therapeutic potential. The overarching goal is to explore the utility of repurposing from a practical standpoint, recognising that this strategy can manifest in various forms. While drug repurposing often refers to the repositioning of off-patent FDA-approved drugs to new clinical indications, it can also include the repositioning of failed and patented drugs. As such, potential repurposing candidates fall into one of the following classes: generic, patented, and failed drugs, each of which have their own associated advantages and constraints [21].

Generic drugs refer to those which are off patent, approved by regulatory agencies, and typically readily available in pharmacies. Due to the extensive testing and pre-clinical and clinical trials which led to their approval, and their monitored use in the population over a long period of time, data relating to their safety and efficacy is both extensive and easily accessible. As a result, generic drugs are typically favoured for repurposing by those in academia, small biotech companies, and not-for-profit research organisations because they are cheaper and lower risk [22].

Patented drugs, which can be extended to include clinical investigational compounds, are patent-protected, and are typically approved or in the late stages of clinical trials. Access to data relating to the safety and efficacy of such compounds is usually limited to their respective pharmaceutical companies which own the patents.

Similarly protected, failed drugs (also called abandoned or discontinued pharmaceutical agents) have been through some stage(s) of clinical trial but did not reach approval, whether due to inadequate efficacy against the intended indication, issues with safety, or lack of funding [23, 24].

About 90% of drugs that move through the clinical trials process do not receive approval and make it to clinical use [25], yielding a significant financial loss for stakeholders in most cases [24]. Repositioning, or ‘rescuing’, such drugs for new indications is therefore particularly attractive to the companies that invest their time and resources on the drugs, only for them to fail at a late stage. As with patented drugs, safety and efficacy data on these failed compounds are not easily accessible to those outside of the company in which they were developed [26]. Other challenges include addressing the reasons for their initial failure to ensure that the same issues do not hinder their success in a new context [27]. Because of limited access to safety and efficacy data and profit concerns, the repurposing of both patented and failed drugs is more attractive to their corresponding pharmaceutical companies that hold the patents than those in academia and other research organisations.

### Strategies to identify efficacious compounds

Recently, efforts have been made to compile comprehensive drug repurposing libraries, providing a centralised list of drugs available for repurposing, often off-patent drugs available as generics [28]. Databases may include additional information for each compound, such as their affected molecular pathways and genes, pharmacology, and side effects [29]. There are numerous ways of determining which drugs in such libraries are likely to be effective against a particular condition; screening methods can be knowledge-based, drug-based, activity-based, in silico or in vitro. While these screening methods are reviewed extensively elsewhere [29–32] we will delve into them briefly in this review because of their paramount significance in the drug repurposing process. We want to emphasise the importance of accurate identification and comprehensive in vitro*/*in vivo testing during the pre-clinical stage, as accurate identification ensures that potential drug candidates are thoroughly evaluated, minimising false leads, and increasing the likelihood of successful translation to clinical trials.

**Fig. 2:**
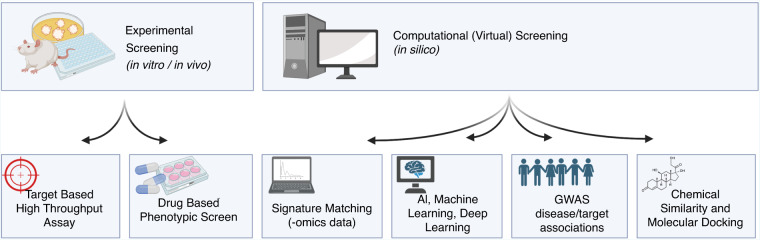
Potential screening methods for identifying efficacious repurposed drugs. These methods may either *be* in vitro or in vivo studies, and may include drug-based phenotypic screens, target-based high throughput assays. Alternatively, computational or virtual screening (in silico) may be done through signature matching (using –omics data), AI or machine and deep learning, genome-wide association studies and disease/target association studies, or chemical similarity and molecular docking simulations. Created using Biorender, February 2023.

Generally, experimental approaches for drug discovery are classified as either target-based or drug-based (Fig. 2). In target-based screening, researchers investigate the interaction between drugs and specific well-defined molecular targets, often using cell-based assays [33]. On the other hand, drug-based (phenotypic) screening relies on cellular or disease models to assess drug effects based on phenotypic outcomes like cell viability and proliferation [34]. Both methods have been used successfully in drug repurposing [32].

One instance of drug repurposing through target-based screening involves Tamoxifen. Initially developed as a contraceptive, after failing to suppress ovulation, it was repurposed for the treatment of breast cancer [35] Researchers later identified the oestrogen receptor (ER) as a potential target in breast cancer, leading to clinical trials evaluating Tamoxifen’s efficacy in blocking the ER. It was found to effectively inhibit oestrogen and slow the growth of ER+ breast cancers [36] and since became one of the most widely used and effective drugs in the treatment of this type of cancer [37]. In contrast, Auranofin which was originally an anti-arthritic medication, offers a drug-based screening example for the treatment of gastrointestinal stromal tumours (GIST) [38, 39]. Its use was discovered through a drug-based phenotypic screen for cell proliferation, Pesetto et. al., revealed that auranofin can effectively and selectively target GIST cells, including those which are resistant to standard treatments such as imatinib [38].

In the age of –omics and big data, computational methods of repurposed drug screening are becoming increasingly favourable [40]. In signature matching screens, the proteomic, metabolomic, and genomic signatures of cancer cells can be compared with those of drug-treated cells, allowing the prediction of which drugs are most likely to be effective against the pathology by reversing dysregulation and restoring a healthy –omics profile [26]. This in silico screening method has been demonstrated in the identification of cimetidine, an anti-peptic ulcer drug, as a potential treatment for lung adenocarcinoma [41]. Serota et. al., subsequently experimentally validated this prediction in vitro and in vivo, showing the drug was able to inhibit lung tumour cells in mouse xenograft models.

Genome-wide association studies (GWAS) have also been used to highlight genetic variants associated with certain diseases, and therefore potential therapeutic targets [42]. A repurposed drug may then be investigated for the treatment of the disease if it is known to target the protein or pathway identified in the GWAS. A recent study by Lin et. al., identified the anti-psychotic imipramine for the treatment of glioblastoma (GB) using this method [42]. They found that imipramine-targeted GB cells have a higher sensitivity than to temozolomide (TMZ), the current standard chemotherapy for this tumour.

Artificial intelligence, machine learning, and deep learning can also be used to uncover potential repurposing candidates. In some cases, text mining can be performed to find new associations between drugs and diseases [43]. A recent text mining study [44], uses PubMed literature to study cancer metastasis-related genes and identify repurposed drugs that may target them. Detroja et. al., demonstrate that aspirin can be repurposed to target TP53 and curcumin for MMP9, both targets strongly associated with cancer metastasis [44]. Using similar text mining methods, clinical observations can be analysed to find links between diseases and drug repositioning opportunities [45, 46]. For example, numerous systematic reviews have investigated the association between metformin and lower incidence of cancer, leading to its current use for a variety of cancers [47–49].

Investigating chemical similarity between drugs and performing molecular docking simulations represent yet another avenue for the identification of drug repurposing candidates. These kinds of analyses require well-validated targets, making it difficult for conditions that are not well understood on a molecular level. Drug screening using molecular docking and dynamics simulations has been used to investigate the possible extension of FDA-approved chemotherapy drugs to treat other types of cancers [50]. The study by Shaikh et. al., uses a virtual screen that measures the interaction of numerous approved drugs with 18 structurally similar kinases important in a variety of cancers [50], shows that thalidomide which is used for treating multiple myeloma, has a good binding potential with both wild-type farnesyltransferase and thymidylate synthase, kinases important to multiple signalling pathways in colon and renal cancer, respectively. This virtual screening technique may be particularly useful for rapid, high-throughput screening to identify drugs that bind to well-defined molecular targets.

A more systematic approach for identifying drugs for repurposing may ameliorate some bias associated with the previously retrospective nature of repurposed drug identification [51]. Even so, extensive and comprehensive in vitro and in vivo validation experiments should be performed in order to fully assess the effect of the drug, to ensure a greater likelihood of success in clinical trials [52].

## Repurposed drugs for cancer treatment

### Clinical applications

Repurposed drugs have diverse clinical applications in cancer therapy including; monotherapy, multi-modal or combination therapy, adverse effect management and chemo/radio sensitisation. They may also be used as prophylactic chemo-preventative agents for at-risk populations, and as adjuvant treatments to prevent recurrence.

Monotherapy involves the utilisation of drugs that possess specific mechanisms of action, enabling them to effectively inhibit tumour growth or induce cancer cell death when administered as standalone treatments. This approach has been employed in the treatment of solid cancers such as glioma. In the case of glioma, a number of repurposed drugs are currently undergoing testing to assess their potential in targeting cancer stem cells (CSCs) in the hopes of increasing survival of glioma patients [53, 54].

Multimodal therapy uses combinations of medications so that practitioners can target different aspects of a complex condition and provide more comprehensive and effective treatment [55]. This approach recognises that complex health conditions are multifaceted, and a single treatment modality may not be sufficient to address all aspects of the problem [56, 57]. The rationale lies in the potential to achieve enhanced treatment outcomes by leveraging complementary mechanisms of action and targeting different aspects of cancer cells or their microenvironment. By combining drugs, researchers aim to maximise therapeutic efficacy, overcome resistance mechanisms, and improve overall patient outcomes [56, 57].

Repurposed drugs may also be used either to reduce recurrence and metastasis, or to prevent development of cancer in at-risk patients [58]. Aggressive cytotoxic treatment for patients with low-risk cancer – such as small and low-grade cancers, may lower their quality of life. Therefore, well-tolerated therapies such as curcumin are being investigated for their ability to prevent cancer progression of patients with low-risk prostate cancer (NCT03769766) [59]. Additionally, metformin is being assessed for its effect on preventing recurrence of endometrial cancer (NCT05192850) and prevention in those considered at-risk of developing breast cancer (NCT01905046) [49].

While personalised treatment currently shows great promise in the treatment of cancer, its widespread implementation faces the formidable challenge of comprehensively profiling the genetic mutations of each patient’s tumour [60]. This process is resource-intensive and often impractical for a significant portion of cancer cases [61]. However, an alternative strategy emerges: the development of a select group of drugs designed to target the most prevalent or critical proteins and pathways in a wide range of cancers. This approach holds the potential to impact a larger patient population with relatively manageable effort.

Current treatments of cancer are associated with varying degrees of treatment failure, manifesting as loco-regional recurrence and/or distant metastasis. Although partial or complete tumour regression can be achieved it can be followed by cancer relapse in many cases, due to the expansion of the CSC population [62] Cancer metastasis and treatment resistance have been purported as the main cause of a number of cancer-related deaths [63, 64].

### Rationale for combination therapy in oncology

As our knowledge of cancer biology continues to expand with discoveries such as inter- and intra-tumoural heterogeneity, and the complex interplay between tumours and their microenvironment, the importance of combination therapies to target multiple pathways simultaneously is increasingly evident [65]. The diversity of genetic, epigenetic, proteomic, and metabolomic alterations demonstrate the variety of the outcomes linked to cancer. Such variety implicates the dysregulation of multiple signalling pathways, even in one tumour [65]. In addition to the tumour itself, it’s essential to consider the dynamic tumour microenvironment (TME). The TME is made up of various cellular and non-cellular components, all interconnected by numerous pathways facilitating communication among cancer cells, CSCs, and the surrounding microenvironment. These pathways include interactions with components of the immune system and complex signalling pathways such as the paracrine Renin-Angiotensin System (RAS), Notch, Wnt/β-catenin, and Sonic Hedgehog [66]. Therefore, a more effective treatment for cancer may require a multi-target strategy, *in lieu* of the long-standing pursuit of a single target ‘silver bullet’ approach [67].

The rationale for combinatory therapy in cancer is based on hallmarks of oncogenesis; the polygenic mutational basis for most malignancies [68], tumour recurrence, metastasis, and the development of resistance to single-agent therapies—including specific targeted therapies [68]. Targeted approaches using monotherapy against specific signalling pathways have shown limited efficacy [69]. Therefore, an urgent need to design alternative combinatorial strategies to replace conventional regimens exists [70].

This approach, while biologically favourable, often results in increased costs of clinical care due to the use of multiple drugs, particularly when the proposed individual treatments (such as on patent drugs) are already prohibitively expensive [56]. However, the use of repurposed drugs makes this strategy considerably more accessible, affordable, and efficient [54]. By utilising these more economical alternatives, combination therapy becomes a more viable option for reducing the overall cost of combinatory cancer treatments [71].

It has been shown that drug combination therapy with two to three drugs each with a unique mode of action may overcome challenges relating to efficacy [72]. For example, the discovery of thalidomide’s anti-angiogenic properties led to a ground-breaking initial clinical trial on patients with recurrent/refractory multiple myeloma [8]. Initial interest in preclinical and clinical trials of thalidomide as a treatment for multiple myeloma was prompted by the historic response rate of 25% in this patient population, who had no other therapeutic alternative [8]. Thalidomide’s clinical efficacy was then validated, with response rates ranging from 25% to 35% [8]. The use of thalidomide in combination with other drugs that are efficacious against myeloma cells was then investigated in subsequent trials which demonstrated response rate of about 50% when combined with steroids, and about 70% when combined with steroids and an alkylating agent such as melphalan [8]. One current example is the testing of thalidomide in combination with chemotherapy, specifically the GDPT regimen, for the treatment of T-cell lymphoma in ongoing clinical trials (NCT01664975) [73]. Furthermore, certain drugs are being individually investigated for their radio-sensitising effect. For instance, nelfinavir has been utilised to enhance the efficacy of standard chemoradiotherapy in the treatment of cervical carcinoma, aiming to increase treatment outcomes (NCT03256916) [74].

These findings of increased efficacy may be due to synergistic effects; if each drug acts on a separate target or signalling pathway, the use of multiple drugs can have a synergistic impact that lowers the required therapeutic dosage for each individual drug [75]. Combination therapy therefore may provide cytotoxic effects on cancer cells while simultaneously reducing their harmful effects on normal cells [76]. Interestingly, a repurposed drug may also possess previously unidentified molecular mechanisms that allow interactions with pathways characteristically involved in the cancer. Essentially, the drugs’ so-called “off-target” effects, may have unanticipated anti-cancer benefits. For example, when identifying off-target effects of etomoxir it was discovered that carnitine palmitoyltransferase I (CPT1) is essential for cancer cell proliferation, and occurs independently of β-oxidation [77]. This suggests that one such function of CPT1 maybe importing long chain fatty acids into the mitochondria for anabolic fates, rather than catabolic oxidation which may support cancer cell proliferation independent of fatty acid oxidation.

**Fig. 3:**
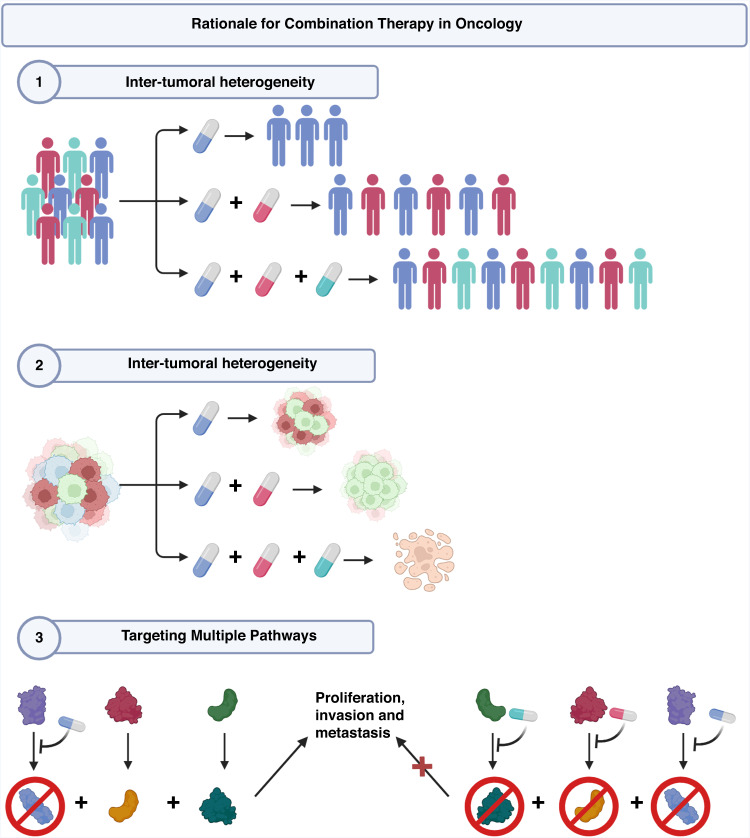
Rationale for combination therapy in oncology. The use of multiple drugs can target the inter-tumoural heterogeneity between patients, intra-tumoural heterogeneity within the same tumour (including the cancer stem cell subpopulation). Multiple drugs also target multiple pathways which may be redundant or compensatory allowing full cessation of proliferation, invasion and metastasis. Created using Biorender, May 2023.

Ultimately, if a disease (such as cancer and psychiatric illnesses) exhibits polygenicity or includes intricate biological signalling networks and feedback loops, inhibition of a single target is typically insufficient to generate the maximum therapeutic impact (Fig. 3). As a result, treating these complex or “smart” malignancies requires “smart” treatment strategies that directly target the expanded oncogenic signalling network. This cannot be achieved by blocking a single protein, but rather through pharmacological inhibition of numerous targets simultaneously. This can have cumulative and even partial effects that outweigh those of single target inhibition [78], thereby overcoming drug resistance.

### Preclinical and clinical trials of repurposed drugs for oncology

Several existing drugs such as niclosamide, metformin, chloroquine and thioridazine have been identified as candidate CSC inhibitors [79]. Thioridazine, an anti-psychotic drug, selectively targets neoplastic cells, and impairs human somatic CSCs capable of initiating leukaemic disease in vivo while having no effect on normal blood stem cells [80, 81]. Metformin has been shown to both eradicate and radiosensitise cancer cells, as well as eradicating radioresistant CSCs by activating AMPK and suppression of mTOR [82]. Metformin is also preferentially cytotoxic to CSCs or CSC-like cells relative to non-CSCs [82]. Disulfiram (DSF) also targets CSCs, by blocking transcriptional availability of CSC signature genes such as Hoxa, Hoxb and Meis1 [83, 84]. Anti-psychotics have shown promise in cancer through inhibition of BHC110/LSD1 which decreases tumour progression. Some anti-psychotics such as brexpiprazole may decrease tumour progression through the downregulation of survivin [85]. Anti- inflammatory drugs (NSAIDs), through various mechanisms have also been used in the treatment of cancer. It has been suggested that up to 20% of all cancer types arise from a chronic inflammatory disease [86]. NSAIDs, in particular, COX-2 inhibitors have shown promising anti-cancer activity in previous laboratory and clinical studies [87–89]. However, its dosage, treatment regimen, risks, and benefits need to be further clarified for their use in cancer.

These pre-clinical and limited clinical trials show that drug repurposing for cancer treatment offers a promising approach to developing new cancer therapies that are both safe and cost-effective. However, while there have been a number of repurposed drugs that have entered clinical trials for cancer treatment, they are often tested as monotherapy, rather than in combination with other drugs [90–92]. This is, even though combination therapy with multiple drugs has been shown to be an effective approach in cancer treatment [72, 93]. The use of multiple drugs with different mechanisms of action, allows simultaneous targeting of multiple pathways involved in cancer development to increase the likelihood of successful treatment outcomes [90, 92, 94].

One reason why there are fewer clinical trials exploring combination therapy with repurposed drugs may be due to the cost and complexity of running clinical trials [8, 21]. Combining multiple drugs in a clinical trial requires careful consideration of dosing, timing, and potential interactions between the drugs, which can be difficult to coordinate [8, 90]. Additionally, repurposed drugs may have different mechanisms of action and side effect profiles, which can make it challenging to determine the optimal combination for a given patient population [91]. Combinatory investigations should not only encompass assessments of efficacy and safety but also delve into the exploration of optimal dosages and synergistic effects with other treatments before they’re taken further in clinical trials. This comprehensive approach ensures a stronger evidence base for the inclusion of drugs in clinical trials and increases the likelihood of successful therapeutic outcomes.

The recent SCALOP-2 [95] and LU001 [96] clinical trials have underscored the critical need for comprehensive preclinical investigations before repurposing drugs for clinical trials. These trials have revealed challenges associated with certain drug combinations in specific contexts, prompting a more cautious approach to drug repurposing. In the case of the SCALOP-2 trial, the rationale for the inclusion of nelfinavir could be much stronger. The decision to include this drug was primarily based on its potential radiosensitisation effects, supported by preclinical data demonstrating its inhibition of PI3K and Akt phosphorylation [95]. However, a notable gap in the study was the lack of a thorough investigation into drug synergy and rigorous preclinical assessment of dosages for this combination, including the use of organoids or other non-human models. Such an approach could have provided a more robust foundation for supporting nelfinavir’s potential efficacy and safety in clinical trials, particularly considering the prevalence of KRAS mutations in pancreatic cancer. Similarly, in the LU001 trial, the inclusion of metformin for the treatment of locally advanced non-small cell lung cancer raised questions about the strength of its rationale. The primary basis for including metformin was its known antineoplastic effects observed in epidemiologic and retrospective studies [96]. However, to enhance the credibility of this decision, a more comprehensive preclinical investigation is essential. This should focus on elucidating the precise mechanisms of action of metformin in this cancer and assessing its synergy with concurrent treatments, different dosages of metformin should be explored to identify the most effective and tolerable regimen.

There is a need for more emphasis on running clinical trials for repurposed drugs in combination with other drugs for cancer treatment, as combination therapy has the potential to be more effective than single-agent therapy [72]. There have been some successful clinical trials exploring combination therapy with repurposed drugs—for example the CUSP9v3 regimen, a cocktail of nine drugs involved in the coordinated undermining of survival pathways utilised in GB [97]. This research is promising and is moving forward to phase 2 clinical trials [97]. Similarly, a phase I clinical trial on GB using a cocktail of seven repurposed drugs that inhibit the RAS and its related pathways has shown that the treatment is safe and well-tolerated with a median overall survival of 19.9 months [98], However, further research is needed in this area to fully realise the potential of repurposed drugs in cancer treatment. Conducting comprehensive preclinical investigations before advancing combinatory drugs into clinical trials is essential. In the preclinical phase, researchers should not only evaluate the efficacy and safety of drug combinations but also explore different dosages and regimens. This includes assessing the dose-response relationship to determine the optimal dosage for achieving therapeutic effects while minimising adverse effects. Repurposed drugs which have been undergone clinical trials for a new indication are summarised in Table 1.

**Table 1:** List of repurposed drugs that have gone or are undergoing clinical trials for anti- cancer indications.

Highest Phase of Clinical Trials	Drug	Original Indication	Reported Targets	Cancer Types
Pre-clinical	Cimetidine	Reduce stomach acid [127]	H_2_ receptor antagonist	Lung adenocarcinoma [41]
	Clomiphene citrate	Luteal phase dysfunction [128]	Oestrogen agonist	GB [129]
	Etomoxir	Chronic heart failure, Diabetes mellitus [130]	Inhibitor of CPT1, prevent FAO	Bladder cancer [130]
Phase I	Imipramine	Depression [131]	Serotonin receptor, glutamate receptors	Breast cancer [132] NCT03122444 GB [42]
	Thioridazine	Anti-psychotic [133]	D_R_D_2_	AML [80] NCT02096289
	Repaglinide	Diabetes mellitus [134]	Inhibits potassium efflux	Prostate cancer [135] NCT04664725 Ovarian cancer NCT04718740
Phase II	Quinacrine	Malaria, giardiasis, rheumatoid arthritis [90]	p53, FACT-CK2-p53 axis	Colorectal adenocarcinoma NCT01844076 NSSLC [136] NCT01839955
	Auranofin	Arthritis [38]	TrxR	CLL [137] NCT01419691 Ovarian cancer NCT03456700
	Itraconazole	Anti-fungal agent [90]	mTOR-cholesterol trafficking, VDAC1, PDGF-Akt–mTOR axis	NSSLC [138] NCT03664115 BCC and other skin cancers NCT01108094
	Niclosamide	Anti-helminthic drug [90]	Wnt/β-catenin, STAT3, NF-κB, Notch, ROS, mTORC1	Prostate cancer NCT02807805 Colorectal cancer NCT02519582 [139]
	Disulfiram	NSAID, alcohol-aversion drug [90]	ALDH, NAD^+^-dependent proteins	NSSLC [140] NCT00312819 GB NCT01907165
	Diclofenac	NSAID, analgesia [141]	cGMP system, COX-1/2,	BCC NCT01358045 [142]
	Chlorpromazine	Anti-psychotic [143]	D_R_D_2_ agonist PI3K/mTOR	GB [144] NCT04224441
	Lovastatin	Hypercholesterolaemia [145, 146]	HMG-CoA reductase, inhibits some RAS isoprenylation	Ovarian cancer [147] NCT00585052
Phase III	Chloroquine	Malaria, rheumatoid arthritis [90]	Autophagy, PPT1	GB [148] NCT00224978 Breast cancer NCT02333890
	Nelfinavir	HIV [149]	Autophagy and apoptosis, HIV protease	Cervical carcinoma NCT03256916 [74]
	Curcumin	Dermatological diseases [150]	hTERT, Wnt/β-catenin, cytokines, Hippo/YAP	Prostate cancer NCT03769766 Colon cancer [151] NCT00295035
	Genistein	Menopause, osteoporosis, obesity [152]	hTERT, Wnt/β-catenin, cAMP/PKA	Prostate cancer NCT00584532 Bladder cancer NCT00118040 [153]
	Berberine	Bacterial diarrhoea [90]	Ephrin-B2, MMP-2/MMP-9, EMT, miR-101, VEGF	Colorectal adenoma [154] NCT02226185 NCT03281096
	Mebendazole	Intestinal helminthiasis [90]	Chk2, Nbs1, PARP-1, DHODH	Colon cancer NCT03925662 [155]
	Aspirin	NSAID for pain and fever [90]	COX-1/2, ANXA1- NF–κB axis, CDX2, COMMD1–RelA axis	Colon cancer NCT02467582 [156]
	Propranolol	Anti-hypertensive [157]	β-adrenoceptor antagonist	Malignant melanoma [158] NCT02962947
Phase IV	Ritonavir	HIV [90]	p53, CDKs-RB axis, AKT-E2F-1-RB axis	Kaposi’s sarcoma NCT00444379 [159]
	Thalidomide	Sedative, anti-emetic [90]	Proangiogenic factors, VEGF receptor, NF-κB	T-cell Lymphoma NCT01664975 Multiple myeloma NCT00652041 Prostate cancer [160] NCT00020085
	Metformin	Type II diabetes mellitus [90]	AMPK, PI3K-mTOR pathways, BACH1	Prostate Cancer NCT02511665 Breast cancer NCT05507398 NCT01905046 Endometrial carcinoma [49] NCT05192850

Drugs are grouped based on their highest phase of clinical trial reached for their new repurposed indication, and includes details of the drugs original indication, potential molecular targets and examples of ongoing clinical trials/research. Table of Pre-Clinical and Clinical Trials of Repurposed Drugs for Oncology.

*AML* acute myeloid leukaemia, *BCC* basal cell carcinoma, *CLL* chronic lymphocytic leukaemia, *CML* chronic myeloid leukaemia, *EMT* epithelial-to-mesenchymal transition, *FAO* fatty acid oxidation, *GB* glioblastoma, *HIV* human immunodeficiency virus, *NSAID* non-steroidal anti-inflammatory drug, *ROS* reactive oxygen species, *VEGF* vascular endothelial growth factor.

## Barriers to drug repurposing

### Intellectual property and profit concerns

Despite the potential benefits it holds, drug repurposing has not gained much momentum. One of the main reasons for this slower uptake is the presence of various barriers, particularly those related to financial aspects, that have not yet been effectively addressed [99]. The existing business model of pharmaceutical companies plays a significant role in this situation, as they heavily rely on the returns generated from investments in research, development, and marketing of new drugs once they are approved for clinical use [21]. Typically, these financial returns are achieved by obtaining market exclusivity for their drugs, which allows them to market the drugs at high prices without facing competition [100]. When a drug receives approval from the FDA, there are two types of market exclusivity that can be sought: patent protection and statutory and regulatory market exclusivity [99]

To acquire patent-protection, products must generate new intellectual property (IP), whether it be the drug composition itself, the use of the drug for a new indication, reformulation, dosing, or in combination with other treatments [99]. As a repurposed drug is not a new chemical entity and its structure is known, a novel patent claim to the active pharmaceutical ingredient is not possible [101]. For repurposed drugs ‘Use’ patents may be filed to protect the ‘method of use’ of the drug for the new indication, however, use patents are typically weaker than composition-of-matter patents and are therefore more costly for the companies to enforce [99]. Use patents also do not prevent off-label prescriptions, in which medications are prescribed for indications or populations for which they have no regulatory approval for [102]. In the case of drug repurposing, this can mean prescription of the generic version of a drug instead of the more expensive patented alternative which has gone through the regulatory approval process for the new indication and achieved market exclusivity. To tackle this problem pharmaceutical companies are legally prohibited from advertising off-label indications, although physicians are able to prescribe its off-label uses, supported by evidence of their efficacy in it new indications [103] which may take away from potential profits.

Pharmaceutical companies can use alternative methods to exclude other firms from the market when applying for regulatory approval. In the USA, this form of market exclusivity that typically lasts for five years for a new chemical entity, seven years for an orphan drug, and only three years for new indications of existing drugs [99]. The most common pathway to achieving this exclusivity for new indications utilises previous pharmacology and toxicology studies and only requires new information regarding safety and efficacy for the new indication [99]. Since the orphan drug act was introduced in the USA in 1983, providing a longer period of market exclusivity for drugs treating rare diseases, the number of approved treatments has soared from 38 to now over 350 treatments for a variety of rare diseases [104]. Developing incentives to support pharmaceutical companies to invest in drug repurposing, such as extended periods of market exclusivity, may encourage research focus and collaboration in the area [105]. Numerous policy changes including tax breaks and FDA-priority review vouchers have been suggested previously to incentivise R&D efforts into drug repurposing [105, 106], however, how effective these incentives are is unclear due to possible off-label prescription. Some identify the funding of phase II and III clinical trials as the primary incentive problem for drug repurposing [99]. Instead of incentives such as granting market exclusivity, tax breaks, prise funds, or FDA-priority review vouchers [106], funding of these clinical trials could work as a better incentive for drug repurposing research.

It has been reported that the likelihood of the FDA granting approval for a new indication for a drug is highest within 1–2 years of approval for its original indication, before generic entry [107]. For pharmaceutical companies, time and resources may be better invested in the repurposing of failed and patented drugs mentioned previously, as this may optimise the role of financial incentives for pharmaceutical companies on drug repurposing [40]. Those that are not wholly constrained by their business model, whether it be academia, biotech companies, or not-for-profit organisations, are better suited to research into repurposing generic drugs, where available data on efficacy, pharmacology, and safety is extensive [21].

Despite big pharma’s central role in the drug development ecosystem, existing evidence supporting the use of off-patent medications in cancer treatment has been largely due to academic and independent research [105]. However, acquiring and retaining marketing exclusivity requires specialised IP knowledge and resources not typically available for researchers and organisations outside the pharmaceutical industry [99]. Collaborations between academia and pharmaceutical industry are therefore becoming increasingly attractive for drug repurposing projects, as they can help to ameliorate profit-driven limitations of pharmaceutical companies inbuilt into their business model, as well as offer academia expertise in the drug development field [21]. For example, because of collaboration between numerous pharmaceutical companies and the British Medical Research Council, 70 failed drugs were made available for repurposing efforts in 2014 [22]. In the USA, a collaboration between academia, the pharmaceutical industry, and national institutes of health made 58 failed compounds available to academia for repurposing [22]. These collaborations are promising in this developing field, although some argue that collaborations are limited by extensive negotiation of technology transfer, data access, and IP rights [108]. However, as open-access data and collaboration become more common in the post-COVID-19 climate [109], efficient drug repurposing may be closer on the horizon.

To facilitate drug development for neglected or rare diseases, there are several options available from an IP perspective [110], including patent pools, open licensing, and allowing academic institutions and staff to participate in patent ownership for new medical uses [110, 111]. In addition, new funding models are emerging that involve venture capitals, public funding, and non-for-profit organisations. These models have the potential to greatly impact certain fields of medicine, such as rare disorders, where drug repurposing plays an important role. Together, these options and models are fostering collaboration among stakeholders and facilitating the development of new treatments for neglected and rare diseases [111, 112].

### Biases affecting drug repurposing

Traditionally, repurposed drugs have been identified in retrospective observational studies, which may be subject to immortal time bias and selection bias [51]. In this method, long-term studies which follow patients through life-long treatment are analysed to find associations between drug use and cancer incidence. Such long-term observational studies are particularly subject to immortal time bias leading to an often overestimation of advantages for the treatment group [113]. This bias has been observed in numerous studies of metformin and the incidence of cancer [48], calling into question the reliability of retrospective observational studies in identifying new repurposing candidates. Additionally, when drugs are first tested or used for their original indication, the group(s) being assessed are typically not considered ‘healthy’. For example, the cohort studies of metformin used to identify it as a possible chemotherapeutic were done using patients with diabetes mellitus [47, 48, 114] and because the participants from the original study (diabetics) are different from that for the new indication (cancer patients), a selection bias is created [115]. The biases affecting retrospective observational studies highlights the importance of a more systematic approach to target identification [51], integrating methods covered previously.

### The difficulty of combination therapy

Due to the polygenic mutational basis of cancer, single agent therapies have been historically unsatisfactory in their effect on tumour growth and recurrence rate due to resistance, leading to their use in combination [116]. Approvals for combination therapy are typically based on randomised phase II or III clinical trials which show improved survival compared to the established standard of care [52]. By nature, combination drug trials are more difficult than those for monotherapy, requiring a meticulous study design to accurately reflect the intricacy, efficacy and usefulness of the therapy [117]. The substantial cost, time, and resources involved in clinical trials means that drugs are typically not brought forward to phase III clinical trials if they are ineffective as single agents in phase II trials [51]. To determine if drugs have at least some level of single-agent activity, they require testing in randomised trials as opposed to the more common uncontrolled phase II studies in oncology. These randomised trials would require a large sample size to reflect a potentially small effect, posing a significant financial burden [51] This is further complicated by the low rates of patient participation in clinical trials [71], in which 40% of cancer trials fail due to insufficient patient accrual [118]. This demonstrates how clinical trials are not optimised for the evaluation of combination therapies [52]. Drugs which lack single agent activity may still have a significant effect in combination therapy, making them difficult to eliminate from further evaluation after ‘unsuccessful’ monotherapy phase II clinical trials [51].

The issues mentioned above highlight the importance of comprehensive, reliable, and predictive pre-clinical combination studies for successful clinical translation [52] so that only the most effective therapeutic combinations will be trialled in phase II or III studies, negating unnecessary costs [119]. It has also been suggested that high quality mechanistic evidence of a drug(s) mechanism of action should be obtained before moving repurposed drugs to clinical trial [52, 120] which may prevent the use of time and resources on potentially unsuccessful and unnecessary clinical trials. Additionally, looking into the molecular basis of synergy in combination therapy would allow for optimisation of drug combinations [119]. Developing high-throughput methods of screening on effective pre-clinical models remains a key target in the pharmaceutical arena, allowing for the thorough investigation of multiple drug’s efficacy and molecular mechanism before moving to clinical trials [121]. For example, recent advances have been made in the creation of a high-throughput in vitro model of the human lung epithelial cell layer which can be used for drug screening [122]. It is also worth noting the importance of pre-clinical models of cancer that are simple enough for screening, yet able to recapitulate the drug response in patients—such as emerging 3D tumour organoids [123]. A study by Movia et al., designed a co-cultured model of the human lung epithelium and show that it can mimic drug resistance mechanisms reflected that in vivo, and are not present in simpler mono-layer culture models [123]. This once again demonstrates the importance of simplistic yet accurate cancer models for comprehensive preclinical trials.

## Conclusions and perspectives

Cancer drugs have become increasingly expensive and prohibitive, with the average cost of a year’s treatment now exceeding $100,000 per annum, while offering only modest improvements in patient survival in most instances. Expensive cancer drugs are a burden on society in two ways: they impose high costs on those funding treatment (patients/insurance/state), and they stifle the development of equally effective but more affordable alternatives. The need for less costly alternatives is particularly dire in cases where the benefit of new therapies is marginal, as the cost-effectiveness ratio is often unfavourable. The high cost of cancer drugs is therefore unsustainable, and innovative solutions are urgently needed to address this burgeoning issue.

There are several potential benefits of drug repurposing for cancer with combination therapies; less toxicity, greater effectiveness, reduced dosage at an equivalent or higher level of effectiveness [119], and the potential to combat drug resistance in current cancer therapies [124]. Furthermore, contrary to de novo development, drug repurposing is a cost-effective and time-saving method for increasing the number of clinically available cancer treatments [30]. The pursuit of a select group of drugs designed to target the most frequently mutated or pivotal proteins and pathways in cancer represents a pragmatic approach to enhance treatment outcomes. By casting a wider net that covers a substantial portion of cancer cases, this strategy offers the promise of more accessible and effective cancer therapies. While personalised treatment remains an important avenue of research [121], the development of such broadly applicable drug combinations can significantly extend our ability to impact the lives of cancer patients. Furthermore, we anticipate that drug repurposing will be a key strategy in the prevention of cancer in at-risk but otherwise healthy population, an issue which is becoming an increasingly important public health concern. The testing of combination therapy, which would focus on the numerous compromised cellular pathways, would be more appropriate for many cancer patients [125]. However, with very few exceptions [126], the pharmaceutical research and testing process is not designed to assist the testing of combination therapies [8].

Collaborations between multiple entities, such as philanthropists, governments/states, not-for-profit organisations, dedicated institutions, initiatives and funds, can generate the necessary funding to support cost-effective clinical trials. The notion that research groups should exclusively focus on discovering “superior” therapies and disregard more affordable alternatives is no longer valid, primarily due to the exorbitant prices of novel cancer drugs. Ultimately, the current high cost of novel cancer drugs is unsustainable, especially when the benefits of these new therapies are minimal at best. In such cases, the cost-effectiveness ratio of these drugs is unfavourable, emphasising the pressing need for less expensive alternatives like drug repurposing.
